# Opioid Intoxication to Withdrawal: A Case of Takotsubo Cardiomyopathy

**DOI:** 10.7759/cureus.59484

**Published:** 2024-05-01

**Authors:** Kashaf Aqeel Zaidi, Parikshit Chapagain, Asef Mahmud

**Affiliations:** 1 Internal Medicine, AdventHealth Orlando, Orlando, USA; 2 Internal Medicine/Pediatrics, AdventHealth Orlando, Orlando, USA

**Keywords:** acute kidney injury, morphine toxicity, opioid dependence, opioid withdrawal, acute heart failure, takotsubo cardiomyopathy

## Abstract

Takotsubo cardiomyopathy (TTC) is characterized by a transient reduction in left ventricular systolic function with apical akinesis. TTC is usually associated with stress and emotional responses; however, opioid withdrawal has been identified as a rare cause of precipitation of TTC. We describe the case of an elderly female with chronic opioid dependence, who presented with symptoms of toxicity and developed TTC upon opioid withdrawal. Her symptoms improved with clonidine. In the time of an ongoing opioid crisis and an attempt to reduce opioid use among patients, this case reinforces the importance of anticipating TTC as a possibly life-threatening complication of sudden discontinuation of opioids in patients who have developed dependence on it.

## Introduction

Takotsubo cardiomyopathy (TTC) is characterized by a transient reduction in left ventricular (LV) systolic function with akinesis of the LV mid-apical segments [1,2]. The prevalence of TTC is estimated to be approximately 0.02% of all hospitalizations in the United States and 1%-2.5% among patients with ST-elevation infarcts and the occurrence is higher in postmenopausal women [3,4]. The diagnosis is based on clinical symptoms, characteristic ventricular dyskinesia and ballooning, new onset EKG abnormalities, elevation of cardiac enzymes in the absence of coronary occlusion, pheochromocytoma, or myocarditis [5]. The exact mechanism of this condition is still being studied, however, the primary hypothesis for pathophysiology is a high catecholamine surge resulting in microvascular coronary spasm or direct injury to the myocardial tissue [6]. While it is associated with benign conditions like stress and emotional changes, TTC can also occur in response to medications that increase the sympathetic drive or withdrawal from medications like opioids and benzodiazepines. Opioid withdrawal is identified as a hyperadrenergic state and with the ongoing opioid dependence crises, it is imperative for clinicians to understand the possible effects of withdrawing opioids in patients with chronic use to prevent potential complications [7]. We present a case of TTC precipitated by opioid withdrawal in a patient who had chronic opioid dependence and initially presented with morphine intoxication.

## Case presentation

A 73-year-old female presented to the emergency department with jerking movements of her extremities for two weeks, and twitching movements of her face for one week. According to the patient’s son, she had also been increasingly confused for approximately one week. The patient and her son denied any fevers, chills, loss of consciousness, or other seizure-like activity but did endorse her having nausea, vomiting, diarrhea, and decreased appetite over the last 2 weeks. She had a past medical history of coronary artery disease status post coronary artery bypass grafting, essential hypertension, chronic kidney disease (CKD) stage 3A with baseline creatinine 1.5, rheumatoid arthritis, obstructive sleep apnea, and long-term opioid dependence. The patient was following up with a pain management specialist and a rheumatologist, she had been prescribed extended-release morphine 60 mg three times daily and a combination of oxycodone-acetaminophen 10 mg-325 mg three times daily for uncontrolled joint and back pain. According to the patient, before this regimen, multiple regimens had been attempted but they were up-titrated due to inadequately controlled pain. On arrival, the patient was drowsy and confused. Her vitals were stable with a blood pressure of 141/59 mmHg, pulse rate of 75 beats per minute, temperature of 97.8 degrees Fahrenheit, respiratory rate of 18 breaths per minute, and maintaining saturation on room air. The movement in the extremities was sudden, brief, and jerky with variable frequency but greater intensity in the distal extremities. The facial twitching was also involuntary, intermittent, and spontaneous. Her pupils were pinpoint, symmetrical, and poorly reactive to light. Initial laboratory investigations revealed an elevated creatinine of 2.82 mg/dL, blood urea nitrogen of 84 mg/dL, and mildly elevated white blood cell count at 15.88. Arterial blood gas analysis revealed mild carbon dioxide retention. The urine toxicology screening was positive for opioids. CT scan performed on admission due to altered mental status did not show any abnormality. Blood and urine cultures were drawn which subsequently did not show any growth. An MRI of her brain demonstrated chronic sequelae of small vessel ischemic disease and did not show acute changes. An electroencephalogram was performed which revealed findings consistent with the encephalopathic process with disorganization of the background with excessive theta slowing as well as interspersed delta activity, generalized periodic discharges, and frontocentral sharply contorted delta activity. In the absence of any other etiology, the patient was managed along the lines of morphine toxicity in the setting of acute kidney injury causing impaired renal clearance. She was initiated on intravenous fluids, and opioids were held. Over the next two days, her muscle twitching and jerking movements improved but remained confused intermittently.

**Figure 1:**
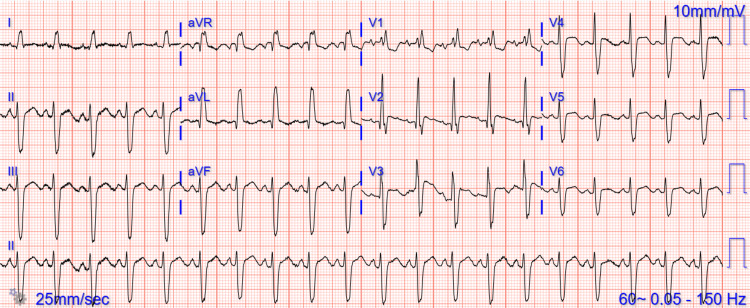
Electrocardiogram showing ST-segment elevations and depressions in various leads.

**Figure 2:**
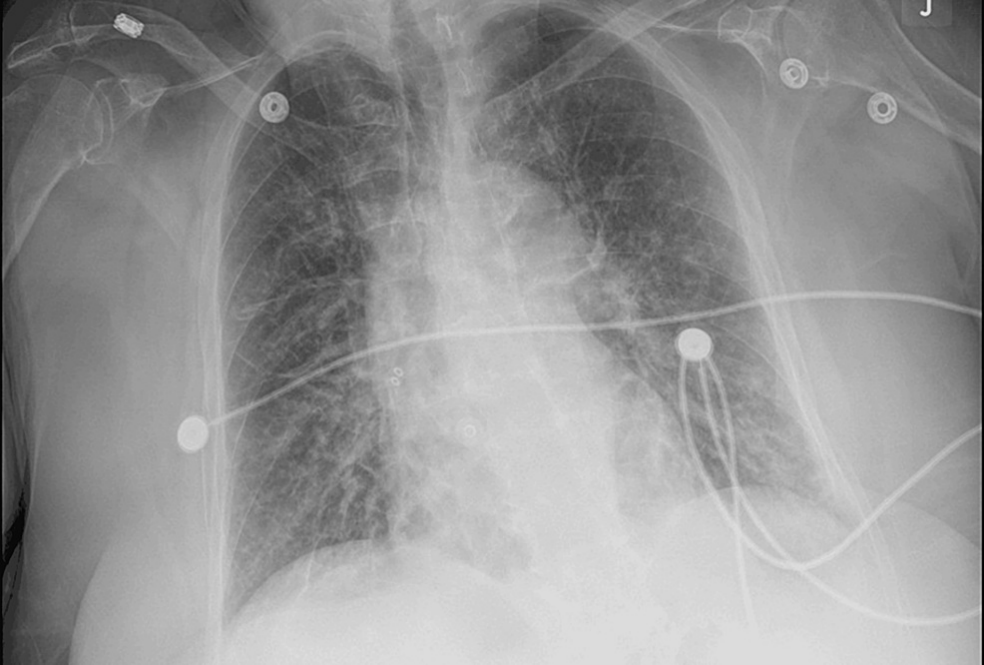
Chest x-ray showing evidence of pulmonary edema.

On Day 3 of admission, she developed acute central chest pain, associated with anxiety, sweating, and shortness of breath with a COWS score of 25 consistent with a moderately severe withdrawal. At this time, her renal function had significantly improved, and her creatinine had returned to baseline. An electrocardiogram revealed new ST-segment changes (Figure 1). A high-sensitivity troponin was checked which was elevated at 439 ng/L compared to 33 ng/L upon admission. A chest x-ray performed revealed findings consistent with pulmonary edema (Figure 2). An echocardiogram was performed, with an ejection fraction of 30%-35%, hypokinesis of the apical anteroseptal, anterior, anterolateral, and inferoseptal myocardium (Video 1); and doppler parameters consistent with elevated ventricular end-diastolic filling pressure.

**Video 1:** Reduced left ventricular (LV) function with akinesis and ballooning of the apex.

An echocardiogram performed 19 months ago showed an ejection fraction of 60-65%, confirming that the change was most likely acute. The patient received nitrates, was started on a heparin infusion, and was transferred to the intensive care unit. She was also started on clonidine for possible opioid withdrawal. The serial troponins and electrocardiograms subsequently improved. Due to her extensive history of coronary artery disease, the patient underwent a left heart catheterization which showed patent bypass grafts with competitive flow; left internal mammary artery to left anterior descending artery, saphenous vein graft to obtuse marginal, and saphenous vein graft to distal right coronary artery. A diagnosis of TTC was established, secondary to opioid withdrawal. The patient was started on sacubitril-valsartan, empagliflozin, metoprolol, clopidogrel, and isosorbide mononitrate. Her overall condition improved and was discharged on goal-directed medical therapy and advised to follow up in the heart failure clinic for the up-titration of these medications for optimal benefit. She was also discharged on a multi-modal pain regimen with a combination of topical diclofenac gel, a short, acute-pain course of oxycodone-acetaminophen, and was advised to follow up with her pain management specialist within three days to discuss the initiation of potential alternative strategies including serotonin-norepinephrine reuptake inhibitors, tricyclic antidepressants, gabapentin, buprenorphine among others, due to her established relationship. The high morphine dose was discontinued due to its potential risk for toxicity.

## Discussion

TTC is a transient condition and most often results in complete recovery of LV systolic function, however, complications such as acute heart failure, fatal ventricular arrhythmias, mitral regurgitation, apical LV thrombosis may occur in the acute phase and can be life-threatening if not identified and managed appropriately [1,2]. By virtue of physician characteristics of nonmaleficence, for patients who are on long-term opioid use for pain management, upon admission to the hospital, the doses are either routinely reduced or held, based on the severity of the patient’s condition. The gradual accumulation of opioids in the patient’s body can lead to myoclonic jerks due to neuroexcitatory effects, similar to our patient. Meperidine and morphine are more commonly associated with myoclonic jerks; however, other opioids can also contribute to it. Sudden administration of naloxone can alleviate the myoclonic jerks but is associated with a risk of withdrawal. For our patient, because the buildup of morphine was thought to be associated with a decreased renal clearance in the setting of acute kidney injury, she was just monitored off of opioids and her myoclonic jerks and mentation continued to improve and vitals were closely monitored for any signs of withdrawal. However, the rapid improvement in her renal function likely contributed to the faster washout of the opioids from her body precipitating withdrawal. Opioid withdrawal can lead to disinhibition of the noradrenergic receptors in the areas of norepinephrine synthesis, resulting in an instant hyperadrenergic surge. This is thought to result in microvascular dysfunction via alpha-1 overstimulation and intracellular calcium overload via excess intracellular calcium influx [6]. Recent studies have shown the successful use of sympatholytic agents in the treatment of withdrawal including clonidine and lofexidine as potential alternatives or adjuncts to long-acting opioids [7,8]. The potential use of these agents for the treatment of TTC in patients with withdrawal is still understudied as there is not enough data on this, as TTC related to opioid withdrawal is still a rare occurrence. Even though there are multiple studies on opioid withdrawal alone, there is not enough data to reflect a case of toxicity leading to withdrawal due to rapid clearance of opioids as acute kidney injury resolves in a patient. So, it is highly recommended to monitor the progression of kidney function when managing opioid toxicity. Mild opioid withdrawal can be managed using methadone or with symptomatic control. Insomnia and anxiety can be managed using benzodiazepines, including temazepam or diazepam. Nausea and vomiting can be addressed with metoclopramide while abdominal pain can be alleviated using hyoscine or propantheline. It is often a difficult decision to determine the acceptable dose of opioids in hospitalized patients to prevent withdrawal, however, a multidisciplinary approach, involving the addiction medicine team can effectively assist with the decision-making process.

## Conclusions

This case reflects on the importance of both the potential toxicity in prescribing opioids, particularly morphine, in patients with CKD and highlights the risk of withdrawal due to rapid improvement in renal function causing rapid clearance. Thus, highlighting the importance of the rate of improvement of kidney function in these patients. As a healthcare worker in the era of the opioid epidemic, it is also crucial to be aware of, and thereby anticipate TTC as a potential complication of opioid withdrawal. While opioid dependence can lead to altered mentation causing serious complications, the process of weaning patients off opioids and the need to reintroduce them appropriately is also crucial. There are no fixed guidelines regarding this; however, a multidisciplinary approach with the involvement of an addiction medicine team and a pain management physician in a non-emergent, outpatient setting, could prove to be beneficial for the patient. The knowledge and hence, anticipation regarding these complications can enable physicians to timely diagnose and manage these patients accordingly to improve patient outcomes. Moreover, this case also emphasizes the importance of alternative pain control strategies for patients with baseline kidney dysfunction.
